# Rheotactic Responses in *Bufo bufo* Tadpoles—Insights Into Adaptation to Lotic Environment

**DOI:** 10.1002/ece3.72800

**Published:** 2025-12-20

**Authors:** Marcel Török‐Oance, Rodica Török‐Oance

**Affiliations:** ^1^ Faculty of Chemistry Biology Geography, Department of Geography West University of Timișoara Timișoara Romania; ^2^ Faculty of Chemistry Biology Geography, Department of Biology West University of Timișoara Timișoara Romania

**Keywords:** behavioural flexibility, *Bufo bufo*, orientation, rheotaxis, structure from motion, tadpoles, water current

## Abstract

Current flow is an important stimulus for amphibian larvae. The common toad ( *Bufo bufo* ) typically breeds in stagnant waters or, less commonly, in slow‐flowing rivers, where tadpoles further develop. This study is the first investigation of *B. bufo* tadpole orientation under varying flow conditions within their natural habitat. Using Structure from Motion techniques, high‐resolution orthophotomosaics were generated for three stream sectors with differing flow velocities, enabling accurate measurements of the orientation of a very large number of tadpoles. Circular statistics were employed to examine tadpoles' orientation in relation to water flow, stream banks, and microtopography. We found that in stagnant water, tadpoles exhibited random orientation in open areas but showed perpendicular alignment to the shoreline near margins. In flowing conditions, tadpoles exhibited positive rheotaxis, more pronounced with increasing current velocity, and were able to maintain their position against the flow despite their typical adaptation to lentic habitats. Additionally, tadpoles manifested the tendency to shelter behind the shores' irregularities and boulders, where flow is reduced, reflecting adaptive strategies to mitigate displacement. This study highlights the ability of *B. bufo* tadpoles to actively respond to flow dynamics, suggesting an adaptive behaviour to cope with the current. Given the tadpoles' absolute dependence on water, behavioural flexibility is a crucial factor for population survival, especially in the context of climate change, where stagnant aquatic habitats are drying up more frequently than rivers. This adaptability could play a key role in enabling tadpoles to exploit more lotic environments, providing a potential resilience mechanism in response to breeding habitat loss.

## Graphical abstract

Current flow is an important stimulus to which tadpoles may react differently. We assessed behaviour and orientation of common toad tadpoles in their natural habitat, in relation to water current. Tadpoles exhibited random orientation in still water and orientation against current in flowing water, suggesting adaptive behaviour to changing habitat conditions. Behaviour flexibility is essential for population survival, especially in the actual context of increasing environmental changes affecting aquatic habitats.

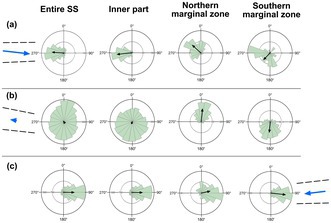

## 1 Introduction

Current flow represents an important stimulus for anuran tadpoles (Schmidt et al. 2011), which may be subjected to current flow ranging from low, limited disturbances, as in standing waters, to stronger currents, as in running waters (Brown and Simmons 2016). How an organism responds to water flow affects its travel time, path, energy use, and the likelihood of reaching its goal (McLaren et al. 2014). Animals that perform goal‐directed swimming movements are likely to have developed mechanisms that allow them to take advantage of favourable flow directions or to cope with adverse currents (Chapman et al. 2011). Rheotaxis represents the capacity of an organism to respond to a current by orienting its body with respect to it (Cherif et al. 2024; Painter 2021) and may be one of the earliest evolutionary mechanisms that allow organisms to favourably interact with their fluid environment (Tolstenkov et al. 2025). Rheotaxis permits organisms to maintain their position (Painter 2021) and to preserve their energy while resisting being shifted by the flow (Coombs et al. 2020). It has been observed in diverse species (Bak‐Coleman et al. 2013). It is widespread in fish (Arnold 1974) and has been recorded in bacteria (Marcos et al. 2012), protozoa (Ricci et al. 1999), jellyfish (Fossette et al. 2015), worms (Bau et al. 2015), amphibians (Durand and Parzefall 1987), etc. Several studies have also examined the orientation of tadpoles in flowing water (Rocha et al. 2002; Simmons et al. 2004; Schmidt et al. 2011; Brown and Simmons 2016); however, to our knowledge, none have specifically addressed species of the genus *Bufo*. A single study based only on visual observations and centred mainly on the description and aggregation behaviour reported that tadpoles of *Rhinella rubescens*, which at the time was placed in the genus *Bufo*, faced upstream (Eterovick and Sazima 1999).

This is the first study focused on the orientation behaviour of common toad ( *Bufo bufo* ) tadpoles in their growth habitat in relation to water current, and, to our knowledge, it represents the first quantitative assessment and analysis of orientation behaviour in the tadpoles of a *Bufo* species. The common toad usually prefers breeding in ponds that are not susceptible to desiccation (Griffiths 1997). However, climate change is increasingly altering hydrological regimes (Blaustein et al. 2010), potentially forcing amphibians to shift oviposition sites to avoid the desiccation risk during offspring development (Beever et al. 2017). In such contexts, the ability to exploit alternative breeding habitats, such as streams or other flowing waters, may become critical. Rheotaxis may function as an adaptive mechanism that enables tadpoles to survive and develop in lotic environments (Eterovick and Sazima 1999) with important ecological significance. This behavioural response could be of particular relevance for common toad populations in regions where traditional lentic breeding habitats, such as ponds and ephemeral pools, are in decline. Species that can exhibit behavioural flexibility in response to environmental changes are more likely to survive (Rymer et al. 2013). This is especially important during the larval phase, considering that the larval period is a critical stage in the life history of toads (Waldman 1982), as tadpoles are more vulnerable than adults (Denver et al. 2002) and larval survival is essential for the continuity of the anuran adult population in terrestrial habitats (Melo et al. 2018).

In this study, we investigate the orientation of common toad tadpoles within their native aquatic habitat, represented by a stream inside Peștera cu Apă Cave from Anina Mountains (Romania), under different water flow conditions. We hypothesise that common toad tadpoles, essentially adapted to lentic environments, respond behaviourally to water current and adjust their orientation in flowing water to cope with hydrodynamic forces. Addressing this knowledge gap is essential for understanding spatial behaviour and ecological adaptation of the tadpoles in lotic environments.

## 2 Methods

### 2.1 Study Area

The study was conducted in a stream running through Peștera cu Apă Cave from Gârliștei Gorges (45°9.89′ N and 21°51.21′ E), located in the Anina Mountains, Romania. Its use as a breeding site by *B. bufo* was first documented in 2021 (Török‐Oance and Török‐Oance 2023), and this is the 2nd year of consecutive observed egg deposition. The toads selected egg deposition locations within the first 60 m from the cave entrance, where the water was almost stagnant or had reduced flow velocity. However, once the tadpoles became mobile, they dispersed throughout the entire downstream sector, also being driven by the water current. The stream presents sectors with flowing water and almost standing water, thus permitting the comparative study of larval orientation in different hydrological conditions. The investigated stream sector (SS) is characterized by narrow widths (0.45–1.8 m), crystal‐clear, shallow water (1.8–18.5 cm in depth) and absence of vegetation.

### 2.2 Survey Methods

Field surveys were conducted in 2022, with the first‐laid eggs observed on May 9. Photographs for analysis were taken when tadpoles were free‐swimming and feeding independently, corresponding to Gosner stage 26. All the photographs were taken on June 14, during daytime. Subsequently, the river began to dry up, turning into a series of disconnected stagnant water pools, which made it impossible to further evaluate the behavior of the tadpoles in flowing water during more advanced developmental stages. By July 17, the water had completely dried up, resulting in the death of the tadpoles.

For analysis, we selected three SSs with different flow velocities. The first sector (SS1) was located 15 m from the cave entrance, the second sector (SS2) was located 45 m from the cave entrance, and the third sector (SS3) was located deeper, at 56 m from the cave entrance. Water temperature, flow velocity, and water depth were measured for each SS. Additionally, we measured flow velocity in two locations where we observed that tadpoles were dragged downstream by the current (at 10 and 30 m from the cave entrance), to determine the water speeds at which tadpoles could no longer maintain their position.

The water temperature was recorded using a Hama T‐350 digital thermometer with an accuracy of 0.1°C. The water depth was measured using a tape metre, and the water flow velocity was determined by the float method (Dobriyal et al. 2017) following the methodology described in Török‐Oance and Török‐Oance (2023).

This study was strictly observational and involved only photo recordings of tadpoles. The study was conducted without contact or manipulation of animals, and no experimental protocols were performed. In this context, no ethical approval was required.

### 2.3 Structure From Motion (SfM) and GIS

We utilised a novel method, SfM, which offers the advantages of accurate recording of a very large number of tadpoles across a larger scale habitat than a regular photo. SfM, widely utilised in geosciences, has recently gained popularity in biological and ecological research. For example, it has been applied to make morphological measurements of marine vertebrates (Hodgson et al. 2020), to map habitats and nest occupancy in gentoo penguin colonies (McDowall and Lynch 2017), to render underwater environments for seagrass restoration (Ventura et al. 2022), and to analyse various marine ecosystems (Bayley and Mogg 2020; Heres et al. 2024).

Many studies on larval orientation rely on photographs or videos, capturing limited areas and fewer individuals, often in artificial settings, more simplified than natural habitats (Brown and Simmons 2016; Katz et al. 1981; Schmidt et al. 2011; Wassersug et al. 1981). The result of the SfM method is an orthophotomosaic, which is a highly accurate composite aerial image created by stitching together a series of individual aerial photos. Using simple photographs as a data source, the SfM workflow produces a 3D dense point cloud, from which a high spatial resolution Digital Elevation Model (DEM) and an orthophotomosaic can be generated (Fonstad et al. 2013). The process reconstructs real‐world scenes and simultaneously records the position of individuals within the terrain (McDowall and Lynch 2017).

Digital photographs were collected with a Panasonic Lumix DMC‐FZ1000 camera (20.1 megapixel resolution), using a film equivalent focal length of 24 mm. We captured a varying number of images for each SS, depending on the size of the area covered. Specifically, the orthophotomosaics were generated using 36 images for SS1, 47 images for SS2, and 62 images for SS3. Photos were taken all around the SS from various locations, at heights between 1 and 1.4 m, with 60% overlap. Two Godox LED lamps were oriented toward the cave walls, producing a low‐intensity, diffuse light that allowed better focusing of the camera and enabled the operator to move without a headlamp. The presence of light slightly disturbed the tadpoles; although they did not change their orientation, they became more active. Therefore, before taking the photographs, we waited 20 min for the tadpoles' recovery, a duration considered long enough for tadpoles to become quiescent (Breden et al. 1982; Wassersug et al. 1981). A flash was used during photography, but it did not appear to affect tadpoles' behaviour, probably due to the extremely short exposure time (approximately 5 ms). Flash photography has previously been used successfully to study larval orientation in darkness (Katz et al. 1981). Care was taken to ensure that the operator's movements did not disturb the tadpoles while taking the photographs, a task facilitated by the spacious cave gallery.

Due to the lack of GPS signal inside the cave, four coded targets have been placed in the corners of each scene to achieve accurate geometrical results. The distances between the targets were precisely measured using a SNDWAY T40 laser metre (accuracy of ±2 mm). These targets were automatically detected in Agisoft Metashape software as reference points and were used to generate the scale of the model, enabling the production of a very high‐resolution model (Verma and Bourke 2019).

The workflow was performed in Agisoft Metashape Professional v1.4 (Agisoft LLC, 2018) software and followed successive steps: aligning photos, building the dense point cloud, building the 3D polygonal model, generating texture, building the tiled model, building the DEM and the orthophotomosaic. Finally, each orthophotomosaic was referenced in a local cartesian coordinate system, assuming arbitrarily that the north direction corresponds to the top of the orthophotomosaic.

The orthophotomosaics were imported into ArcGIS Pro v3.2 (ESRI, USA, 2023) and manually digitised, extracting tadpoles as vectors. The digitising of tadpoles was performed from tail to snout so that the orientation of the resulting vectors matched their orientation. When the density of tadpoles was too high to distinguish each one precisely, some were not digitised. These situations were encountered especially in SS2, in the marginal zones, over small areas of just a few square centimetres. Although we cannot discern the orientation of these tadpoles in the photos, we noticed on‐site that they all had approximately the same orientation, specifically toward the shore. Therefore, even if it had been possible to measure their orientation, this would not have influenced the result of the analysis. The orientation for each tadpole was calculated clockwise in degrees, from 0° to 360°, using the line bearing function in ArcGIS Pro v3.2.

We observed that tadpoles tended to cluster near the stream banks, a phenomenon referred to as edge effect (Katz et al. 1981), which could potentially introduce bias into our results. To address this, we separately analysed tadpoles near the riverbanks. We established a 3 cm buffer zone for each bank, extending inward from the stream bank within SS, which will be referred to as marginal zone. According to Katz et al. (1981) and supported by our field observation, this distance represents the range where the edge effect is noticeable.

The stream bank sinuosity coefficient (Sc) was calculated for each bank of the SSs as the ratio between the length of the stream bank and the straight‐line distance between its two endpoints (Zăvoianu 1985). The higher the value of Sc, the higher the shore's sinuosity.

The water surface area within each SS was measured in ArcGIS, and the density of the tadpoles, expressed as the number of tadpoles per square metre, was calculated.

### 2.4 Statistical Analysis

A statistical analysis was conducted on the orientation of all digitised tadpoles within each SS. In addition, for each SS, the orientation of the tadpoles located specifically in the marginal zones was analysed separately. Finally, the orientation of only the tadpoles from the inner part of each SS was analysed, excluding those located within the marginal zones.

Angles measuring tadpoles' orientations are cyclic, known in statistics as circular variables (Batschelet 1981). Thus, we used circular statistics to analyze tadpoles' orientation in relation to water flow direction and stream bank orientation. The Rayleigh *Z* test was performed to determine whether tadpoles' orientation significantly deviates from uniformity (randomness) (Landler et al. 2018). This test is based on the mean vector length (*r*), also known as vector strength, which serves as a measure of the concentration of data in a certain region of the circle (Batschelet 1981). The mean vector length ranges from 0 to 1. If most input vectors have similar directions, *r* is close to 1. When input vector directions span the entire compass, *r* is small (near zero). The angle of the mean vector was also calculated, and it represents the mean orientation, indicating the central tendency of the data. The Rayleigh test assumes that any potential deviation from uniformity follows the von Mises distribution (Mardia and Jupp 1999). The von Mises distribution is unimodal and symmetric, being the equivalent in circular statistics for the normal distribution for the linear data, and its dispersion is quantified by the concentration parameter (*k*) (Batschelet 1981). Higher values of *k* indicate greater concentration around the mean direction, while values close to zero suggest uniformity. The ability to reject the null hypothesis of randomness increases as the concentration parameter *k* value increases. Circular standard deviation and 95% confidence interval were computed for each sample. The circular statistic was performed in Oriana v4.02 (Kovach Computing Services, UK, 2013).

## 3 Results

One orthophotomosaic with a spatial resolution of 0.1 mm/pixel was obtained for each analysed river sector using SfM. The largest sector was SS2, with an area of 3.94 m^2^, followed by SS3, with an area of 2.32 m^2^. SS1 was the smallest sector, with an area of only 0.73 m^2^. Since orthophotomosaics also captured part of the riverbanks, the surfaces occupied by water within these sectors were smaller (Table 1).

**TABLE 1:** Environmental features and number of digitised tadpoles in the SSs on the day of photography.

Variable	Stream sectors		
	SS1	SS2	SS3
Distance from cave entrance (m)	15	45	56
Water surface area (m^2^)	0.48	1.77	1.26
Mean water flow velocity (cm/s)	12	0.8	7
Mean water depth (cm)	5.1	7.4	5.5
Water temperature (°C)	12.6	12.3	12
Mean water flow direction (degrees)	93.23	279.35	263.45
Northern bank mean orientation (degrees)	90.99–269.01	101.2–258.8	84.19–275.81
Southern bank mean orientation (degrees)	88.36–271.64	96.23–263.77	88.58–271.42
Northern bank Cs/Southern bank Cs	1.32/1.17	1.28/1.34	1.17/1.29
Number of digitised tadpoles	784	6516	8830
Tadpoles' density (tadpoles/m^2^)	1633	3681	7008

The SSs were characterised by a relatively constant water temperature and similar average water depths (Table 1). Although illumination differed among the SSs (SS1 receiving natural light, SS2 having natural light barely perceptible to the human eye, and SS3 being dark), the use of identical artificial light sources allowed us to consider that illumination was consistent across all SSs at the time of photography. We also assumed that nutrient input was consistent across all SSs, given that cave environments are characterised by limited trophic resources (Culver and Pipan 2009), as they lack primary producers and photosynthesis (Moldovan et al. 2018) and food resources are mainly transported by the stream, the three analysed sectors being situated within a 40‐m distance. So, the relevant environmental factors were almost identical across the three SSs, except for water flow velocity, which varied, and we can consider that larval orientation is essentially influenced by water flow velocity.

**FIGURE 1:**
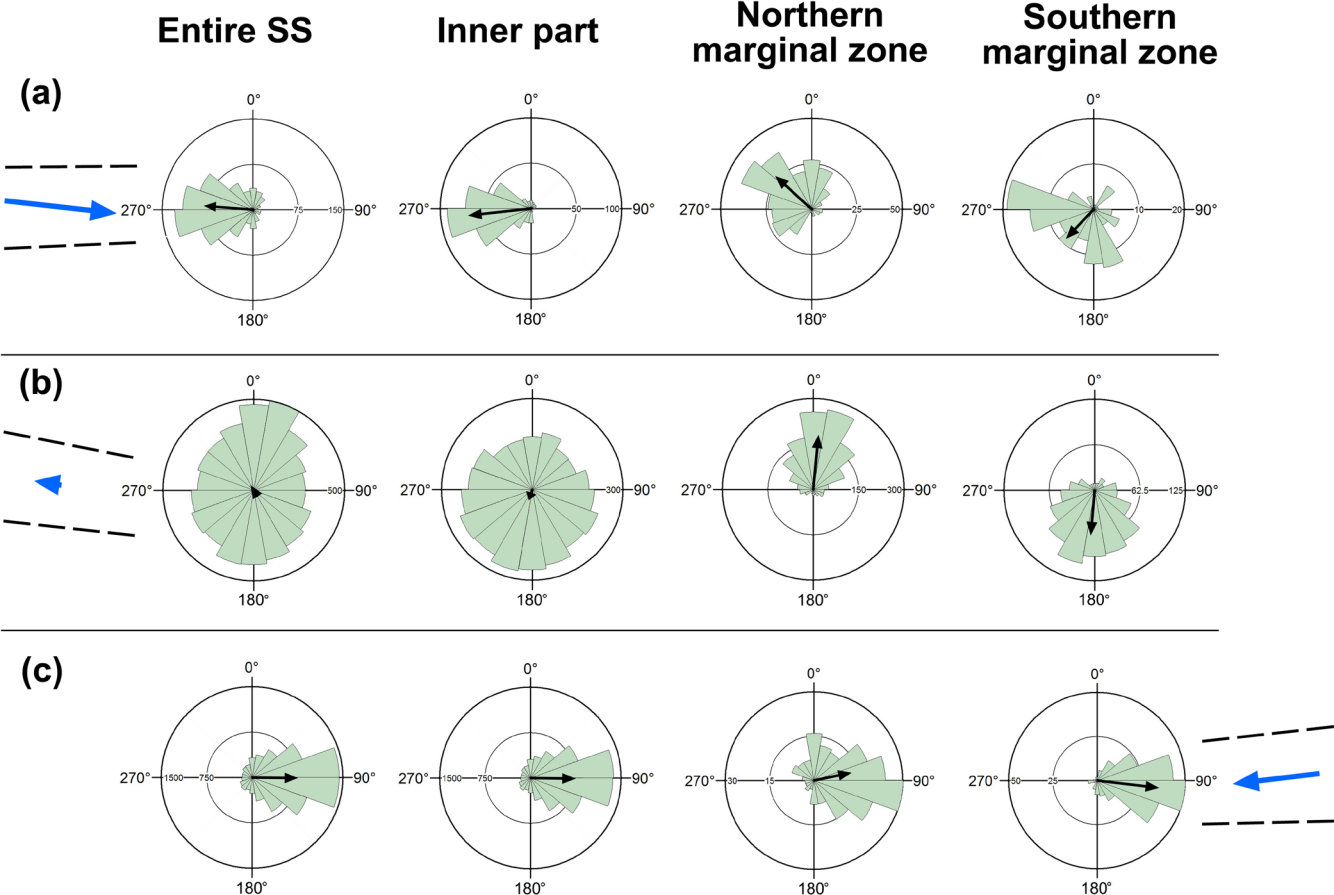
Rose diagrams showing *Bufo bufo* tadpoles' orientation in relation to water flow using a binwidth of 20°. The length of the bars illustrates the number of tadpoles. (a) SS1, (b) SS2, (c) SS3. The direction of the black arrows indicates the mean orientation of the tadpoles, and the length of the black arrows, the vector strength (*r*). The blue arrows indicate the mean direction of the water current. The higher the length of the arrow, the higher the water flow velocity. The dashed lines indicate the mean direction of the northern and southern banks.

A total number of 16,130 tadpoles were digitised for all three SSs. The orientation of tadpoles in all SSs was not random, except for SS2 (Table 2 and Figure 1).

**TABLE 2:** Results of Rayleigh's Test of uniformity and the circular statistics descriptive variables for the SSs.

Stream sector	Mean orientation (degrees)	Median (degrees)	*r*	Circ. SD (degrees)	*Z*	*p*	*k*	95% CI (degrees)	*N*
**Stream Sector 1 (SS1)**									
SS1	274.69	272	0.52	65.54	211.83	**< 0.0001**	1.21	(269.57, 279.68)	784
SS1 inner	263.83	261.73	0.7	50.41	181.7	**< 0.0001**	1.88	(258.83, 268.84)	394
SS1 NMZ	312.32	314	0.53	64.91	75.91	**< 0.0001**	1.23	(303.89, 320.74)	274
SS1 SMZ	223.23	230	0.42	75.28	20.64	**< 0.0001**	0.93	(206.58, 239.89)	116
**Stream Sector 2 (SS2)**									
SS2	326.68	3	0.02	163.17	1.96	0.141	0.04	(23.44, 269.92)	6516
SS2 inner	195.79	203	0.1	121.97	45.08	**< 0.0001**	0.21	(183.99, 207.6)	4187
SS2 NMZ	5.29	8	0.6	58.6	538.22	**< 0.0001**	1.48	(2.22, 8.35)	1532
SS2 SMZ	185.19	186	0.49	68.27	192.72	**< 0.0001**	1.13	(179.84,190.5)	797
**Stream Sector 3 (SS3)**									
SS3	91.72	91	0.51	66.93	2256.25	**< 0.0001**	1.17	(90.16, 93.27)	8830
SS3 inner	92.08	91	0.51	66.95	2145.63	**< 0.0001**	1.12	(90.49, 93.68)	8403
SS3 NMZ	78.03	82	0.43	75	36.59	**< 0.0001**	0.94	(65.5., 90.53)	203
SS3 SMZ	96.67	98	0.7	48.7	108.78	**< 0.0001**	1.99	(90.29, 103.05)	224

Abbreviations: inner = inner part of the stream sector, NMZ = northern bank marginal zone, SMZ = southern bank marginal zone.

In SS1, the mean orientation of all 784 tadpoles was 274.69°, consistently opposing the water current. The Rayleigh test indicates strong evidence against uniformity (*Z* = 211.83, *N* = 784, *p* < 0.0001). The mean vector length (*r* = 0.52) reveals a clustered location of the tadpoles around the mean orientation, in agreement with the value of *k* (Table 2, Figure 1a). The tadpoles only from the inner part of SS1 showed an even higher concentration around the mean orientation (*r* = 0.7, *k* = 1.88), also against the water current (Figure 1a). The tadpoles within the marginal zones also had a non‐random orientation. The tadpoles in the northern marginal zone had an average orientation of 312.32° (*Z* = 75.91, *N* = 274, *p* < 0.0001) and a high concentration of data around the mean orientation (*r* = 0.53). The tadpoles from the southern marginal zone showed a preferred orientation whose mean value was 223.23° (*Z* = 20.64, *N* = 116, *p* < 0.0001), but a lower concentration of data around the mean orientation (*r* = 0.42) (Figure 1a).

**FIGURE 2:**
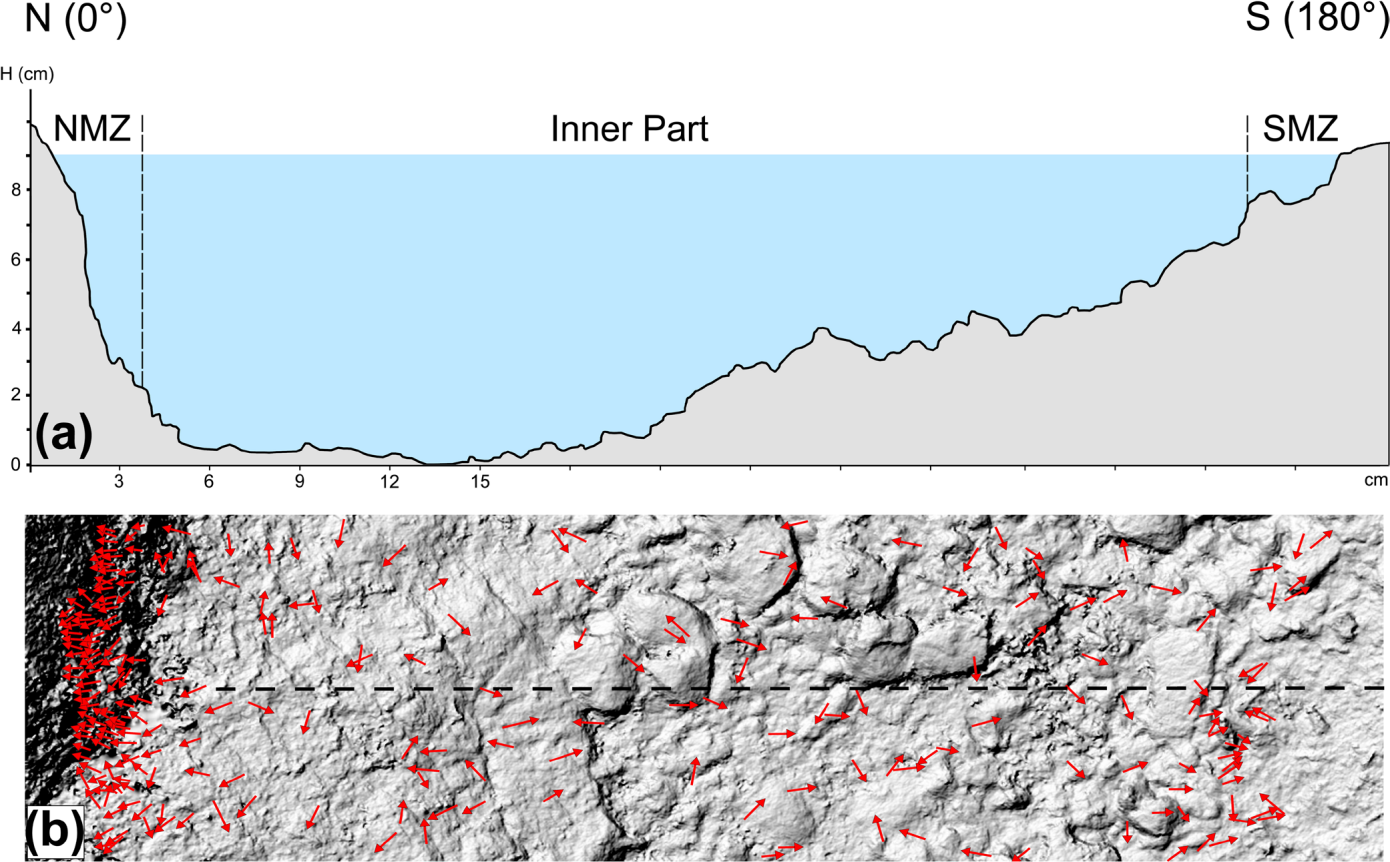
(a) Transverse profile of SS2, derived from DEM, illustrating morphological and water depth differences between the northern marginal zone (NMZ) and the southern marginal zone (SMZ). (b) *Bufo bufo* tadpoles, digitised as vectors within a 5 cm wide area on either side of the profile line (marked by the black dashed line). Arrowheads indicate the direction the tadpoles are facing. In the background, a hillshade DEM provides a visual representation of the microtopography of the streambed.

In SS2, the water was almost stagnant, and the mean water flow direction was 260.66° (Table 1). Globally for SS2, tadpoles showed no consistent directional preference (*Z* = 1.96, *N* = 6516, *p* = 0.141). The mean length vector was nearly zero, which means that tadpoles' orientations span the entire compass (Figure 1b). The low concentration factor (*k* = 0.04) also suggests uniformity. The Rayleigh test for the inner part of SS2 revealed departure from uniformity, but the very low mean vector length (*r* = 0.1) indicated that tadpoles were weakly clustered and nearly randomly oriented, with only a slight directional trend (Figure 1b). In contrast, tadpoles from only the marginal zones predominantly oriented toward the banks (Figure 1b). Near the northern bank, the tadpoles were more concentrated around the mean orientation (*r* = 0.6, *k* = 1.48) than the tadpoles near the southern bank (*r* = 0.49, *k* = 1.13) (Figure 2).

**FIGURE 3:**
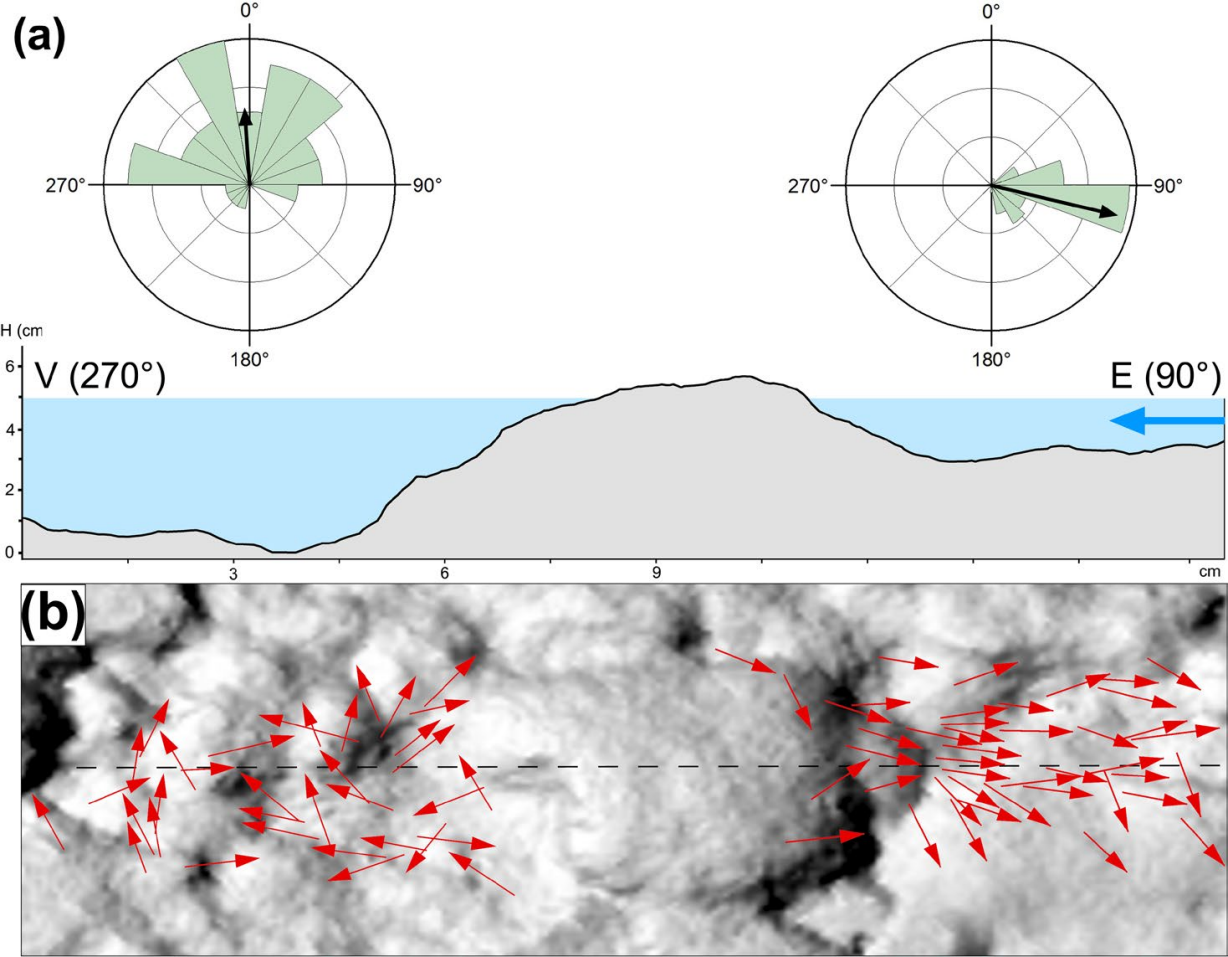
(a) Topographic profile in SS3, derived from DEM, showing a boulder that disrupts water flow. The blue arrow indicates the direction of the current. Rose diagrams illustrate the orientation of tadpoles upstream (right) and downstream (left) of the boulder. Orientation was measured for tadpoles within a buffer zone equal to the boulder's width. (b) Vectors representing *B. bufo* tadpoles overlaid on a hillshade DEM, highlighting fine‐scale topography. Arrowheads indicate the direction the tadpoles are facing. The black dashed line marks the topographic profile line.

In SS3, the water flow velocity was higher than in SS2 but lower than in SS1 (Table 1). The mean water flow direction was 263.45°. In the inner area, the marginal zones, and across the entire SS3, tadpoles predominantly oriented themselves against the water current, with mean orientations ranging between 78° and 92°, indicating a clear directional pattern (Table 2, Figure 1c). In many places, boulders scattered across the streambed disrupted the water current, leading to differences between nearby tadpoles' orientation upstream and downstream of each obstacle (Figure 3). Upstream of the boulder, 91% of tadpoles showed a strongly clustered orientation (mean orientation 104.25°, *r* = 0.91, *Z* = 35.48, *N* = 43, *p* < 0.001, *k* = 5.75). Downstream, tadpoles displayed a different directional pattern (mean orientation 355.87°, *r* = 0.52, *Z* = 9.36, *N* = 35, *p* < 0.001, *k* = 1.21), with only 52% of individuals concentrated around the mean direction and a substantially lower concentration (*k*).

## 4 Discussion

In the standing water sector (SS2), distinct orientation patterns were identified in the inner area and in the marginal zones. In the inner area, the tadpoles were oriented in all cardinal directions and showed no directional preference. However, in the marginal zones, most tadpoles were agglomerated along the shoreline with an orientation toward it. The tadpoles' orientations were perpendicular to the margins and very close to them, consistent with the experimentally observed edge effect in *Xenopus laevis* (Katz et al. 1981). Although tadpoles were generally parallel to one another, this alignment shifted whenever the shoreline changed direction, so that they remained parallel within each margin segment but not across differently oriented segments. This indicates that inter‐larval parallelism is most likely secondary to their contour‐following perpendicular orientation to the shoreline. The polarization of tadpoles along the banks was more pronounced where the water was deeper and the rocky banks were steeper, compared to the margins with shallower water and gentler slopes. In places with deeper water, the edge effect also appeared in depth, with tadpoles positioned perpendicularly to the shore at more depth levels. These suggest the tadpoles' preference for deeper water, which is consistent with observations made by Bardsley and Beebee (1998).

The tadpole alignment along the margins may indicate a wall‐following behaviour. Wall‐following behaviours represent unconditioned behavioural tendencies in relation to walls, including the propensity to move along walls, stay close to walls, or maintain contact with walls or boundaries (Hänzi and Straka 2018; Sharma et al. 2009) and have been described in many animal taxa (Scharf and Farji‐Brener 2024). The biological significance of wall‐following behaviour is largely unknown (Sharma et al. 2009), but it may involve complex functions (Chen et al. 2024) such as spatial orientation, defensive strategies, and exploiting favourable biotic or abiotic conditions (Scharf and Farji‐Brener 2024). The tadpoles' tendency to actively maintain a close vicinity with margins could also confer a foraging benefit. Although cave environments are devoid of vegetation and poor in trophic resources compared to epigean environments (Culver and Pipan 2019), they host microorganisms that inhabit water, sediments and rock surfaces, many of them forming extensive biofilms (Martin‐Pozas et al. 2023). These biofilms develop on various interfaces, including solid‐water interfaces in aquatic environments (Vlasceanu et al. 2000), and represent an important food source for tadpoles (Kloh et al. 2018). At stage 25 Gosner, the two labial tooth rows are fully formed (Bonacci et al. 2008), and the oral apparatus of Bufonidae permits biting and scraping at surfaces, as well as feeding on suspension particles (Harrison 1987). Thus, foraging may have contributed to the observed edge effect. Observations on *Anaxyrus americanus* also showed that tadpoles agglomerated near pond edges and scraped algae and bacteria for feeding (Pearman 1993).

In the running water sectors (SS1 and SS3), more pronounced in the central zones, the majority of tadpoles exhibited robust positive rheotaxis, predominantly orienting with their heads against the current. Despite being adapted to lentic habitats, the tadpoles were able to maintain position against the current, displaying station‐holding behaviour with intense tail movements while oriented upstream. In the marginal zones, where flow was weaker, tadpoles still tended to orient upstream, although less strongly than those in the central areas. Positive rheotaxis can be an avoidance mechanism against being carried away by the current (Wassersug 1973) or against being sucked by fish and amphibians (Roberts et al. 2009). Station‐holding behaviour is one of the three main categories of behaviour associated with rheotaxis, alongside goal‐directed and flow‐refuging behaviours (Coombs et al. 2020). Research on tadpoles' kinematics shows that tadpoles are efficient undulatory swimmers (Wassersug 1997) with mechanical efficiency similar to that of sub‐carangiform fishes of comparable size (Wassersug and Hoff 1985), but with little stamina at rapid velocities (Wassersug and Feder 1983). However, tadpoles of different species distinguish themselves by behavioural differences (Wassersug 1973), and the few studies conducted so far on tadpole rheotaxis indicate various responses. Thus, Simmons et al. (2004) reported positive rheotaxis for tadpoles of *X. laevis* at developmental stages 47–56. A study on *Lithobates catesbeianus* described tadpole movements in flow as a directed random walk (Schmidt et al. 2011). Another research on both *X. laevis* and *L. catesbeianus* revealed that *X. laevis* exhibited strong rheotaxis with clear station‐holding behaviour, whereas bullfrog tadpoles displayed positive rheotaxis with only low to moderate accuracy and high inter‐individual variability (Brown and Simmons 2016).

Tadpoles expressed a more pronounced rheotactic behaviour with increasing water velocity, suggesting that they can perceive differences in flow and react accordingly. However, the ability of tadpoles to maintain position was overcome at higher flow velocities. While most tadpoles still maintained position through rheotaxis at a mean velocity of 12 cm/s, some were carried away by the current. These observations indicate that a mean velocity of 12 cm/s may represent a critical threshold at which their ability to resist the current begins to be exceeded, consistent with experimental results observed in *L. catesbeianus* (Brown and Simmons 2016). This was further supported by our measurements at two locations outside the analysed SSs, where water velocities of 13 and 19 cm/s, respectively, exceeded the tadpoles' ability to maintain position, sweeping them downstream.

Tadpoles also expressed flow refuging behaviour, attempting to escape the stronger central current by swimming toward the bottom and stream margins, where friction with the streambed and banks reduces water velocity (Shaw et al. 2011). The tendency to move toward side walls or even positioning nearby was also noticed in bullfrog tadpoles (Brown and Simmons 2016; Schmidt et al. 2011). Additionally, tadpoles manifested the tendency to shelter behind shoreline irregularities and boulders, where flow is attenuated, reflecting adaptive strategies to mitigate displacement. This behaviour likely underpins their increased abundance in marginal sectors exhibiting pronounced indentations and a higher coefficient of sinuosity, in contrast to straighter‐edged sectors where fewer tadpoles were recorded. In accordance with the heterogeneous flow around obstacles, tadpoles positioned upstream and downstream of boulders showed different orientations, explainable by obstacle‐induced changes in local flow pattern and velocity (Coombs et al. 2020). Thus, while tadpoles upstream of the boulders showed strong rheotaxis, those behind the boulders exhibited an orientation closer to random.

Our findings indicate that the orientation of common toad tadpoles at Gosner stage 26 is strongly related to current. However, as tadpoles mature, their behaviour evolves (Beiswenger 1975), so that tadpoles at different developmental stages may exhibit different behavioural responses. Therefore, further research is needed to explore how tadpoles' orientation and behaviour in relation to current may vary across developmental stages.

The non‐invasive SfM technique provided a comprehensive synoptic image for each SS, capturing a very large number of tadpoles and so enabling strong statistical power to detect subtle patterns. Observing a vast number of tadpoles in their natural habitat, along with detailed microtopography, ensures that the behavior captured is authentic and ecologically relevant, offering insights beyond those obtainable in artificial settings (Kobayashi et al. 2014). This highlights field‐based synoptic imaging as a powerful tool for behavioral ecology analysis. The main limitation of this approach is that the images capture a single moment in time. Although temporal data were limited, consistent spatial patterns and subsequent visual field observations suggest orientation stability of the tadpoles, related to current. Another constraint was the blurring of some moving tadpoles in the orthophotomosaic, which prevented the digitization of approximately 1%–2% of tadpoles, though this had negligible impact given large sample sizes. Finally, the method is restricted to clear water or sub‐aerial environments.

## 5 Conclusions

The research highlights that tadpoles actively respond to water currents, suggesting adaptive behaviour for coping with changing flowing conditions of the aquatic habitat. The ability of tadpoles, typically adapted to lentic habitats, to adjust their orientation and behaviour in flowing water may represent a compensatory mechanism to support the species' persistence in areas where traditional breeding sites, such as ponds or temporary pools, are declining, and where streams, due to their more stable hydrological regime, often represent the only aquatic habitats available. Thus, larval responses to water flow may represent an essential element in species resilience under accelerating climate change and habitat transformation.

## Author Contributions

**Marcel Török‐Oance:** conceptualization (equal), data curation (equal), formal analysis (lead), investigation (equal), methodology (lead), project administration (supporting), resources (lead), visualization (lead), writing – original draft (equal), writing – review and editing (supporting). **Rodica Török‐Oance:** conceptualization (equal), data curation (equal), formal analysis (supporting), investigation (equal), methodology (supporting), project administration (lead), resources (supporting), visualization (supporting), writing – original draft (equal), writing – review and editing (lead).

## Conflicts of Interest

The authors declare no conflicts of interest.

## Data Availability

Data available from Mendeley Data: https://data.mendeley.com/datasets/jnrj2r5v49/1.
